# Tumors of the oral cavity: CO2 laser management

**DOI:** 10.4317/medoral.22811

**Published:** 2018-12-24

**Authors:** Kuauhyama Luna-Ortiz, Sergio C. Hidalgo-Bahena, Tania L. Muñoz-Gutiérrez, Adalberto Mosqueda-Taylor

**Affiliations:** 1 MD. Department of Head and Neck Surgery, Instituto Nacional de Cancerología, Tlalpan Mexico; 2 Department of Head and Neck Surgery, Instituto Nacional de Cancerología, Tlalpan Mexico; 3 DDS. Departamento Atención a la Salud, Universidad Autónoma Metropolitana Xochimilco. Mexico

## Abstract

**Background:**

Cancer of the oral cavity combined with oropharyngeal cancer is the sixth leading cause of death for cancer worldwide. Surgery remains the standard treatment for this disease in early clinical and locally advanced stages. Numerous studies have shown that laser management is useful for premalignant lesions in the oral cavity; however, there is no conclusive evidence that its use is appropriate in cancer of the oral cavity and that results are comparable with traditional surgery. The objective of this study is to determine cancer control after wide local resection with CO2 laser for oral malignant neoplasms.

**Material and Methods:**

Retrospective study in patients with tumors of the oral cavity who were considered for surgical resection with CO2 laser from January 2006-December 2015. Demographic data, treatment modalities, histopathological diagnosis and clinical stage variables were obtained. All resections were done with the use of the microspot. Patients with cancer of the tongue were not included because a specific protocol for these patients does exist in our institution.

**Results:**

There were twenty patients, 10 male and 10 female with a average age of 58 years (range: 20-92 years). Mean age was 53.5 years for females and 63 years for males. Twelve (60%) patients are alive and disease free and four (20%) were lost free of disease.

**Conclusions:**

CO2 laser is an acceptable surgical method for the management of small lesions in the oral cavity. We cannot rule out that small lesions of the oral cavity with positive neck could be managed in this manner, adding treatment to the neck, producing an adequate local regional control. However, this hypothesis requires additional studies.

** Key words:**CO2 Laser, oral cavity, cancer, treatment.

## Introduction

Cancer of the oral cavity in combination with cancer of the oropharynx is the sixth cause of death of cancer worldwide. Despite improvements in management, 5-year survival is only 45−50%. The occurrence of this cancer has been linked mainly to the use of tobacco, betel nut and alcohol consumption. There are different prognostic factors such as the primary site, lymph nodes involved, tumor thickness and surgical margins (1). The standard treatment for this type of cancer is radical surgery and preservation of aesthetics and function as much as possible with wide macroscopic tridimensional margins from 15 mm onwards to obtain a microscopic margin >5 mm (2-4). Surgery can be performed with a traditional scalpel, cryotherapy, or with electrocautery with laser (5-9). Laser has been used in surgery since the 1960s. Advantages include clean margins and decreased damage to the surrounding resected tissue. With assistance of microscope resection is more apparent, which is not the case scalpel or other type of instrument where the resection is greater (10-13). Multiple studies have proven that laser management is adequate in premalignant lesions of the oral cavity (14); however, no conclusive evidence is available that its use is appropriate in cancer of the oral cavity and that the results with its use are comparable to those with traditional surgery. The objective of this study is to determine if wide local resection of cancer of the oral cavity with a CO2 laser offers adequate cancer control.

## Material and Methods

A retrospective study was carried out including patients with tumors of the oral cavity who were considered for surgical resection with the CO2 laser from January 2006−December 2015. Variables obtained were treatment, histopathological diagnosis and clinical stages. All resections were done using microscope and the microspot. This study did not include patients with cancer of the tongue because a protocol for these types of patients already exists in our institution. Survival staging was not established for this group due to the variety of histological diagnoses and prognoses ( Table 1). All patients in our study were treated as outpatients or had a 1-day hospital stay.

**Table 1:**
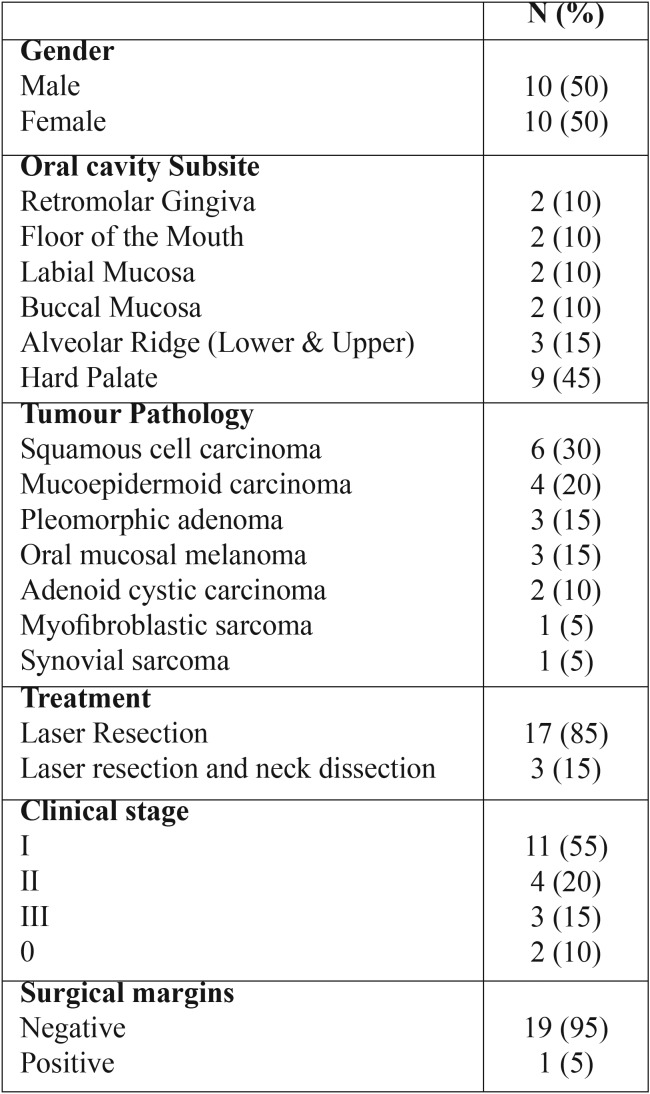
Clinical features.

## Results

Twenty patients were included: 10 males and 10 females with a mean age of 58 years (20−92 years). The mean age for females was 53.5 years and for males was 63 years. Clinical features are shown in T Table 1. T Table 2 shows age, subsite, histologic types of tumors, surgical margins, actual stage (at the time of the study) and follow-up time (in months) for all included patients.

**Table 2:**
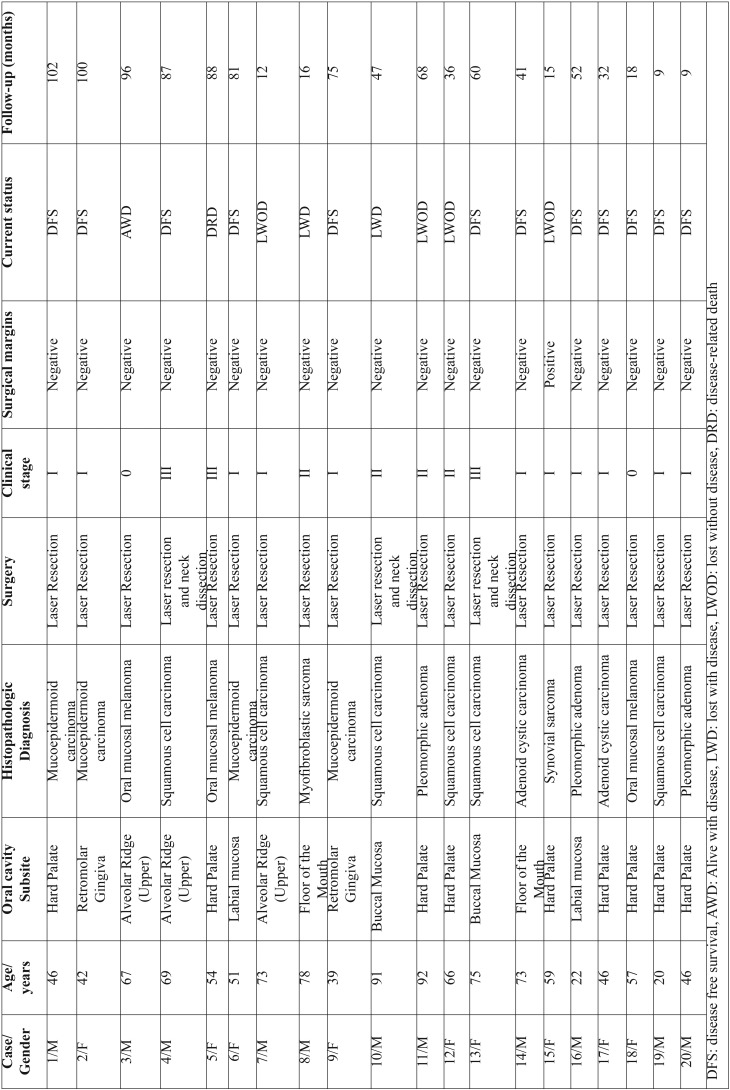
Diagnosis, stage and follow-up.

## Discussion

Management of oral cavity tumors with CO2 laser is not a standard treatment. The existing experience is rather limited and the equipment required to carry out the procedure is expensive, particularly when performed under the microscope. Advantages of surgical treatment with CO2 laser in diseases of the oral cavity include minimal damage to the adjacent tissues, reduction of the acute inflammatory reaction, and decrease in the activity of myofibroblasts which leads to a reduced contraction of tissue in scarring (15). In addition, patients managed with this type of approach have less postoperative pain, as it is a less invasive surgery in which no sutures are used and complex reconstruction using free flaps is not required (16). Tuncer *et al.* (17) demonstrated these advantages where they compared conventional surgery with laser surgery in oral cavity disease. The authors evaluated the effect of thermal damage in regard to the histopathological diagnosis, pain control and postoperative complications and demonstrated that thermal damage does not have an adverse effect with respect to the histopathological diagnosis. There was also better pain control with the use of the laser and there were no intra- or postoperative complications. It is important to emphasize that, in our series, despite having extensive resections, patients only remained hospitalized for 1 day, which is an additional advantage when compared with those who have complex reconstructions along with prolonged hospital stay. Surgery remains as the treatment of choice for cancer of the oral cavity in early clinical stages. Although there is information about the usefulness of the CO2 laser in the treatment of benign and premalignant lesions, there is little evidence to demonstrate if this type of approach is appropriate for management of malignant lesions of the oral cavity, particularly epidermoid carcinoma. However, we present a case series with diverse pathologies that included six cases of epidermoid carcinoma and all patients underwent wide local excision. Only one case had a positive margin in the definitive histopathology; however, this had no impact on recurrence as the patient had no recurrence during follow-up.

Jerjes *et al.* (16) carried out a prospective study to examine results after CO2 laser resection in T1/T2 N0 epidermoid carcinoma in order to determine the disease-free period and survival in 90 patients. The most frequently affected sites in these patients were tongue and floor of the mouth and the most common clinical presentation was as ulcers of the oral mucosa. Eighty-one patients presented with T1N0 and nine patients T2N0. Histopathological analysis revealed that half of the patients had moderately differentiated epidermoid carcinoma, 18 patients moderate to poorly differentiated and 19 patients poorly differentiated carcinoma. Seventy-three patients had complete resection with 11 cases of recurrence (12%). Three-year survival was 86.7%. Twelve patients died during follow-up, nine of whom died due to causes unrelated to the cancer, two due to local regional recurrence and one due to metastatic pulmonary disease. When compared with our series, 95% of our margins were negative; however, in our series the histologies were diverse which are associated with different prognosis. Despite this, 60% of our patients are still alive and free of disease; in addition, there were four patients lost without disease.

Three out of the six cases of epidermoid carcinoma included in our series were treated with selective neck dissection (SND). The first one was managed with CO2 laser resection. At 1-year follow- up, the patient presented with a new lesion. Radical surgery with inferior maxillectomy plus SND at levels I−III was carried out. Histopathological report was adenocarcinoma of the salivary gland and negative lymph nodes. This case may represent a second primary tumor. The second patient underwent laser resection (oral mucosa subsite) plus SND at levels I−III and had one node positive for malignancy. The patient was referred for adjuvant radiotherapy (RT) (72 Gy). This patient was lost to follow-up. The third case was similarly referred for laser excision of the primary tumor with SND at levels I−III and has demonstrated no evidence of recurrence.

Three cases had mucosal melanoma. One presented multiple melanotic lesions during follow-up and were resected on five occasions with CO2 laser with histopathological diagnosis of melanosis. At 8 years of follow-up (2015), metastatic disease in the mediastinum was documented by positron emission tomography. The patient continues with follow-up and is being treated with palliative CT. Another patient with clinical stage III melanoma was referred for laser surgery of melanotic lesions on five occasions. Only the first oral lesion was reported as melanoma. The remainder were reported as melanosis. At 8 years of follow-up the patient presented with recurrence in the palate and neck and required management with total palatectomy and neck dissection, receiving adjuvant RT and CT. The patient presented disease progression to the central nervous system and bone and died of disease. The third patient with melanoma had recurrence of the melanoma and was referred for local excision with CO2 laser. Histopathological diagnosis was melanoma in situ. Currently the patient is reported with hard palate melanosis without signs of malignancy. In our experience, we do not perform surgical excision of melanotic macules and avoid mutilating resections in melanoma of the head and neck because patients do not die from local disease. Local recurrences are the rule in which excisions are done on multiple occasions until the patients finally die due to metastatic disease. Five-year survival is 4.5−29% despite aggressive treatment of the disease (18).

There were nine cases with tumors of the minor salivary glands, three of which corresponded to pleomorphic adenomas, four mucoepidermoid carcinomas and two adenoid cystic carcinomas. There are no previous reports of treatment with CO2 laser for these types of salivary gland tumors; however, in our series all cases have remained disease-free and none was submitted to neck dissection. We previously demonstrated that neck dissection in adenoid cystic carcinoma is only therapeutically useful (19).

The first one had two patients with diagnosis of sarcoma. The first has a myofibroblastic sarcoma of the floor of the mouth and was referred for surgery with laser excision and presented persistence of the disease. The patient did not accept radical treatment. The second patient had a diagnosis of monophasic synovial sarcoma and was referred for laser surgery treatment, and surgical bed (bone) was reported with neoplasm. Radical surgery was decided upon with a partial palatectomy. The histopathological report of the palatectomy was negative for malignancy. There are no reports in the literature for treatment of this type of histology with CO2 laser; however, due to the biology of this type of neoplasm, patient choice must be very selective.

## Conclusions

Evolution of oncology has produced an effect on the decision to perform less invasive surgical procedures. It has been demonstrated that the results of using CO2 laser are in a significative number comparable with those obtained with radical surgery. CO2 laser demonstrates that this is an acceptable surgical method for the management of small lesions of the oral cavity. In fact, we do not rule out the notion that small lesions of the oral cavity with positive neck can be managed in this way, adding treatment to the neck, producing appropriate local regional control. However, the limitation of this work is that it is a retrospective study and further studies are required to better estimate the benefits derived from this type of approach of cancer of the oral and maxillofacial region.

## References

[1] Jerjes W, Upile T, Hamdoon Z, Mosse CA, Akram S, Hopper C (2011). Prospective evaluation outcome after transoral CO2 laser resection of T1/T2 oral squamous cell carcinoma. Oral Surg Oral Med Oral Pathol Oral Radiol Endod.

[2] Neukman FW, Stelzle F (2010). Laser tumor treatment in oral and maxillofacial surgery. Physics Procedia.

[3] Montero PH, Patel S (2015). Cancer of the oral cavity. Surg Oncol Clin North Am.

[4] Tirelli G, Piovesana M, Gatto A, Tofanelli M, Biasotto M, Boscolo Nata F (2015). Narrow band imaging in the intra-operative definition of resection margins in oral cavity and oropharyngeal cancer. Oral Oncol.

[5] Van der Hem PS, Nauta JM, Van der Wal JE, Roodenburg JLN (2005). The results of CO2 laser surgery in patients with oral leukoplakia: a 25 year follow up. Oral Oncol.

[6] Ishii J, Fujita K, Munemoto S, Komori T (2004). Management of oral leukoplakia by laser surgery: relation between recurrence and malignant transformation and clinicopathological features. J Clin Laser Med Surg.

[7] Thomson PJ, Wylie J (2002). Interventional laser surgery: an effective surgical and diagnostic tool in oral precancer management. Int J Oral Maxillofac Surg.

[8] Hamadah O, Thomson PJ (2009). Factors affecting carbon dioxide laser treatment for oral precancer: a patient cohort study. Laser Surg Med.

[9] Ishii J, Fujita K, Komori T (2003). Laser surgery as a treatment for oral leukoplakia. Oral Oncol.

[10] Canis M, Ihler F, Martin A, Ihler F, Martin A (2014). Enoral laser microsurgery for squamous cell carcinoma of the oral cavity. Head Neck.

[11] Durel J, Gaudet J, Kunduk M, McWorter AJ (2010). Transoral laser microsurgery for malignancies of the upper aerodigestive tract. Otorhinolaryngol Clin.

[12] Del Corso G, Gissi DB, Tarsitano A, Costabile E, Marchetti C, Montebugnoli L (2015). Laser evaporation versus laser excision of oral leukoplakia: A retrospective study with long-term follow-up. J Cranio-Maxillo-Facial Surg.

[13] Sinha P, Hackman T, Nussenbaum B, Wu N (2014). Transoral laser microsurgery for oral squamous cell carcinoma: oncologic outcomes and prognostic factors. Head Neck.

[14] Chainani-Wu N, Lee D, Madden E, Sim C, Collins K, Silverman S Jr (2015). Clinical predictors of oral leukoplakia recurrence following CO2 laser vaporization. J Cranio-Maxillo-Facial Surg.

[15] Roodenburg JL, Witjes MJ, de Veld DC, Tan IB, Nauta JM (2002). Lasers in dentristy: use of lasers in oral an maxillofacial surgery. Ned Tijdschr Tandheelkd.

[16] Jerjes W, Hamdoon Z, Hopper C (2012). CO2 lasers in the management of potentially malignant and malignant oral disorders. Head Neck Oncol.

[17] Tuncer I, Özcakir-Tomruk C, Sencift K, Cöloğlu S (2009). Comparison of conventional surgery an CO2 laser on intraoral soft tissue pathologies and evaluation of the collateral thermal damage. Photomed Laser Surg.

[18] Luna-Ortiz K, Campos-Ramos E, Pasche P, Mosqueda-Taylor A (2011). Oral mucosal melanoma: conservative treatment including laser surgery. Med Oral Patol Oral Cir Bucal.

[19] Luna-Ortiz K, Carmona LT, Cano VAM, Mosqueda-Taylor A, Herrera-Gómez A, Villavicencio-Valencia V (2009). Adenoid cystic carcinoma of the tongue clinicopathological study and survival analysis. Head Neck Oncol.

